# HDL-C presents a suggestive negative moderating trend for the association between total cholesterol and colorectal polyps: model construction and bootstrap internal assessment of a predictive nomogram

**DOI:** 10.3389/fendo.2026.1864105

**Published:** 2026-07-22

**Authors:** Lichao Gao, Yujia Liu, Xiaojia Lv, Zhengli Dou

**Affiliations:** The Fourth Affiliated Hospital of Anhui Medical University, Hefei, China

**Keywords:** cholesterol, colorectal polyps, high-density lipoprotein cholesterol, nomogram, risk prediction, suggestive moderating trend

## Abstract

**Background:**

Colorectal polyps are classic precancerous lesions of colorectal cancer, closely linked to lipid metabolic disorders. Elevated total cholesterol does not consistently raise polyp risk, implying interactive effects among lipoprotein fractions. This study aimed to explore whether HDL-C exhibits a moderating trend in the TC-polyp association and construct a predictive nomogram with bootstrap internal performance evaluation.

**Methods:**

A retrospective single-center case-control study enrolling 411 hospitalized colonoscopy patients. Our pre-defined primary multivariate logistic regression was adjusted for age and standardized objective baseline indicators (gender, BMI category, *Helicobacter pylori*, GSP, CST4). Normal weight was set as BMI reference; restricted cubic splines were applied to test BMI nonlinearity. Data on smoking, alcohol intake, diet, physical activity and family history of colorectal polyps were incompletely recorded in inpatient medical records and thus excluded from regression adjustment. Sensitivity analyses substituting non-HDL-C for TC were performed to eliminate compositional coupling bias between TC and HDL-C. Subgroup analyses stratified by polyp pathological subtypes were conducted. Nomogram discrimination and calibration were evaluated via ROC curves and 500 bootstrap resampling on the full cohort without train-test splitting. Random forest was used to rank variable importance; such rankings cannot independently corroborate the TC × HDL-C interaction term.

**Results:**

After adjustment for age and baseline clinical covariates, we detected a suggestive inverse TC × HDL-C interaction (OR = 0.787, 95% CI: 0.626–0.994, P = 0.040). Sensitivity analysis with non-HDL-C reproduced this consistent inverse trend (P = 0.023). Restricted cubic spline modeling indicated no nonlinear correlation between continuous BMI and polyp risk (P for non-linearity=0.348). The internally assessed nomogram achieved an AUC of 0.834 (95% CI: 0.789–0.879) with satisfactory bootstrap calibration. Consistent inverse moderating trends were observed across adenomatous and hyperplastic polyp subgroups at the descriptive level.

**Conclusion:**

We identified a suggestive inverse TC–HDL-C moderating trend after covariate adjustment, which remained robust in non-HDL-C sensitivity analysis. HDL-C exhibited no independent main effect on colorectal polyps, yet it exerted a suggestive negative moderating trend that attenuated the positive association between total cholesterol and colorectal polyps. The internally assessed nomogram provides preliminary reference for individualized colorectal polyp risk stratification. Observations are constrained by inpatient-only recruitment and incomplete lifestyle covariate adjustment; further large community-based cohorts are needed to replicate these findings.

## Introduction

Colorectal polyps are widely recognized as the most critical precancerous lesions of colorectal cancer (1, 2), and their pathogenesis is closely associated with systemic metabolic disorders (3–5). As one of the most common malignant tumors threatening human health, colorectal cancer has shown a marked increase in incidence in recent years (6), with a trend toward younger onset (7). Over 85% of cases arise from adenomatous polyps through the classic “polyp-adenoma-carcinoma” sequence (8, 9). Given the pivotal role of polyps in colorectal carcinogenesis, identifying risk factors for their formation is essential for early cancer prevention (10).

Among these, abnormal lipid metabolism has emerged as a key area of investigation (11). Hypercholesterolemia is widely considered a major metabolic risk factor that promotes intestinal mucosal hyperplasia, chronic inflammation, and abnormal epithelial proliferation, thereby facilitating polyp development (12). However, clinical observations indicate that not all individuals with elevated cholesterol levels develop colorectal polyps (13), and the incidence of polyps varies considerably among populations with similar cholesterol levels (14). This suggests that the relationship between cholesterol and colorectal polyps is not a simple linear one, but rather modulated by complex interactions among multiple lipid components (15). Consequently, a single lipid parameter is insufficient to accurately reflect the metabolic risk associated with colorectal polyps (16).

High-density lipoprotein cholesterol (HDL-C) is widely recognized as a core “protective lipoprotein” essential for maintaining lipid metabolic homeostasis (17). It is involved in multiple physiological processes, including cholesterol reverse transport—which removes excess cholesterol from peripheral tissues—as well as antioxidant and anti-inflammatory responses (18). Accumulating evidence has demonstrated that HDL exerts a significant protective role in the pathogenesis and progression of cardiovascular diseases and various malignancies (19), and its potential protective effect on the intestinal mucosa has also been suggested in relevant studies (20). However, direct, high-quality clinical evidence remains lacking regarding whether HDL-C modulates the association between cholesterol and colorectal polyps, as well as the specific mode and intensity of this regulatory effect (21).

Against this background, the present study analyzed colorectal polyps using clinical data collected from patients who underwent routine colonoscopy, with rigorous statistical methods. This study aimed first to systematically characterize the association between serum cholesterol levels and the occurrence of colorectal polyps. Second, we aimed to explore whether HDL-C exerts a moderating trend on the cholesterol-polyp association and quantify potential modulating intensity via interaction term modeling. Third, the study aimed to construct a predictive nomogram and perform internal assessment of its predictive performance to facilitate individualized risk stratification in clinical practice.

It should be noted that all participants in this study were hospitalized patients receiving colonoscopy for clinical indications, which may introduce selection bias and alter serum metabolic biomarker levels. In addition, adenomatous and hyperplastic polyps arise from distinct biological proliferation pathways; we further conducted stratified subgroup analyses to explore whether lipid regulation differs across polyp pathological subtypes.

## Materials and methods

### Study design and population

This was a retrospective case–control study conducted at the Department of Gastroenterology, the Fourth Affiliated Hospital of Anhui Medical University. The study group consisted of 310 patients hospitalized between January 1, 2021, and January 30, 2025, who underwent colonoscopy and were diagnosed with colorectal polyps. The control group included 101 patients hospitalized during the same period who underwent colonoscopy with normal findings. The study group comprised 219 males and 91 females, with a mean age of 57.42 ± 10.08 years. The control group included 30 males and 71 females, with a mean age of 61.04 ± 13.99 years.

Inclusion criteria: first-time colonoscopy during hospitalization with no history of polypectomy, no use of hypoglycemic or lipid-lowering therapy within the past six months, and no prior Helicobacter pylori eradication treatment. Pathological diagnosis of colorectal polyps was required to be confirmed independently by two associate chief physicians or above through blinded review. Complete clinical records were necessary, including metabolic indicators, H. pylori status, endoscopy findings, and pathology reports.

Exclusion criteria: presence of non-sporadic polyposis syndromes, such as inflammatory bowel disease, intestinal tuberculosis, familial adenomatous polyposis, or Peutz–Jeghers syndrome; severe impairment of cardiac, cerebral, hepatic, or renal function; or use of medications within the preceding six months that could alter lipid metabolism (e.g., statins, fibrates) or exert chemopreventive effects on colorectal polyps (e.g., aspirin or other nonsteroidal anti-inflammatory drugs).

All enrolled patients were hospitalized individuals undergoing colonoscopy due to gastrointestinal complaints rather than community screening crowds, which may lead to selection bias and interfere with the stability of serum lipid indicators.

### Data collection

General clinical data: Gender, age, body mass index (BMI) classification (underweight, normal weight, overweight, obese), history of hypertension, history of diabetes, and Helicobacter pylori infection status (positive or negative).

Metabolic indicators: Total cholesterol (TC), triglycerides (TG), low−density lipoprotein cholesterol (LDL−C), high−density lipoprotein cholesterol (HDL−C), fasting blood glucose (FBG), glycated serum protein (GSP), uric acid (UA), creatinine (Cr), urea, aspartate aminotransferase/alanine aminotransferase ratio (AST/ALT), total bilirubin (TBil), and total bile acids (TBA).

Tumor markers: carcinoembryonic antigen (CEA), cancer antigen 125 (CA125), cancer antigen 153 (CA153), cancer antigen 199 (CA199), cancer antigen 724 (CA724), cysteine protease inhibitor 4 (CST4).

Polyp characteristics: Polyp location was classified as left colon (descending colon, sigmoid colon, and rectum), right colon (cecum and ascending colon), transverse colon (including hepatic and splenic flexures), or multiple (polyps present in two or more segments). Polyp number was categorized as solitary or multiple (two or more polyps). Histologically, polyps were classified as hyperplastic or adenomatous based on pathology reports.

### Statistical analysis

Data were analyzed with SPSS 25.0 and R 4.2.1. Categorical variables were compared by chi-square test; continuous variables were assessed for normality with t-test or Wilcoxon rank-sum test accordingly. Multicollinearity was excluded by VIF < 5 threshold.

Age is a well-documented confounder for both lipid levels and colorectal polyps, so age adjustment **was** pre-specified in our analytical framework from study design. Considering age is a well-established confounder of both lipid metabolism and colorectal polyp risk in existing epidemiological studies, age was forced into the multivariate regression regardless of univariate P-values to eliminate residual age-related confounding. The primary multivariate logistic regression was adjusted for age and complete objective baseline covariates extracted from electronic medical records: gender, BMI category, Helicobacter pylori infection, GSP and CST4. Normal weight, male sex, and H. pylori-negative status were set as reference categories for categorical variables. Restricted cubic splines were applied to test the nonlinear relationship between continuous BMI and polyp risk.

Smoking, alcohol, diet and activity data were collected through clinical inquiry, yet records lacked unified standards and contained frequent missing values. To avoid missing-data bias, these lifestyle variables were not incorporated into regression. To test interaction robustness and exclude compositional coupling bias, sensitivity analyses were performed using non-HDL-C, LDL-C, and the TC/HDL-C ratio in place of TC. Subgroup analyses by polyp pathological subtype were performed.

The TC × HDL-C interaction term was reverse-coded only for nomogram scoring without altering model results. Model discrimination was measured by AUC, and calibration was evaluated via 500 bootstrap repetitions (internal assessment). A random forest model with 500 decision trees ranked predictors by mean decrease in Gini index. Variable importance rankings only reflect predictive weight within this inpatient cohort and cannot independently confirm the presence of the TC × HDL-C moderating trend.

### Model development and internal assessment

Based on the independent predictors identified in the multivariate moderation analysis, a nomogram was constructed using the rms package in R (version 4.2.1). Each predictor was assigned a weighted score proportional to its regression coefficient, and the total score corresponded to the predicted probability of colorectal polyp presence. Model discrimination was assessed by receiver operating characteristic (ROC) curves and quantified by the area under the curve (AUC) with 95% confidence intervals. Calibration was evaluated using bootstrap-corrected calibration curves with 500 resampling repetitions. To further characterize relative variable predictive weight, a random forest model (random Forest package) was trained with 500 trees, and the mean decrease in Gini index was used to rank predictors.

## Results

### Baseline data

In univariate comparison, the control group had a significantly higher mean age than the polyp group (61.04 ± 13.99 vs. 57.42 ± 10.08 years, P = 0.018). Additionally, the two groups differed significantly in gender, BMI classification, hypertension prevalence, and H. pylori infection rate (all P < 0.05), while diabetes prevalence showed no significant difference (P > 0.05) (**Table 1**). This counterintuitive finding is likely multifactorial. First, the marked sex imbalance between groups—predominantly male in the polyp group (70.6%) versus predominantly female in the control group (70.3%)—introduced substantial confounding, as female sex exhibited a strong protective effect in multivariate analysis (OR = 0.14). Second, the inpatient nature of the cohort may have amplified this disparity: control subjects were older hospitalized patients undergoing colonoscopy for various indications, whereas the polyp group included relatively younger individuals with milder complaints. After adjusting for sex and other covariates, age lost statistical significance (OR = 0.989, P = 0.387), confirming that the crude age difference was driven by confounding and selection bias rather than a genuine inverse age–polyp association.

**Table 1:** Comparison of baseline characteristics between the two groups [n(%)].

Index	Study group	Control group	Statistic	P
Age (years)	57.42 ± 10.08	61.04 ± 13.99	-2.405	0.018
Gender			53.475	<0.001
Male	219 (70.6%)	30 (29.7%)		
Female	91 (29.4%)	71 (70.3%)		
Hypertension			5.879	0.015
Yes	131 (42.3%)	29 (28.7%)		
No	179 (57.7%)	72 (71.3%)		
Diabetes mellitus			1.578	0.209
Yes	46 (14.8%)	10 (9.9%)		
No	264 (85.2%)	91 (90.1%)		
Helicobacter pylori			5.889	0.015
Positive	90 (29.0%)	17 (16.8%)		
Negative	220 (71.0%)	84 (83.2%)		
BMI classification			41.788	<0.001
Underweight	8 (2.6%)	19 (18.8%)		
Normal weight	138 (44.5%)	52 (51.5%)		
Overweight	120 (38.7%)	26 (25.7%)		
Obesity	44 (14.2%)	4 (4.0%)		

Although the control group had a significantly higher mean age than the polyp group in univariate comparison (P = 0.018), age lost independent predictive significance after adjusting for gender, BMI category, H. pylori status, GSP and CST4 in the multivariate model (P = 0.387). The crude intergroup age disparity was mediated by confounding clinical covariates rather than reflecting a causal age-related polyp risk.

### Univariate analysis of key metabolic and tumor markers

Univariate analysis revealed significant differences between the polyp and control groups in total cholesterol, triglycerides, LDL-C, fasting blood glucose, uric acid, creatinine, AST/ALT ratio (all P < 0.05), as well as in tumor markers CA125 and CA724 (both P < 0.01). Among lipid parameters, total cholesterol was significantly higher in the polyp group (4.82 ± 0.91 vs. 4.43 ± 1.12 mmol/L, P = 0.001), whereas HDL-C did not differ significantly between groups (P = 0.495). Glycated serum protein (GSP) and CST4 showed borderline associations (P = 0.156 and P = 0.064, respectively) and were retained for multivariate analysis based on clinical relevance. Complete univariate comparisons of metabolic indicators and tumor markers are provided in **Supplementary Tables 1**, **S2**, respectively. Stratified analyses grouped by polyp location, polyp number and pathological subtype are displayed in **Supplementary Tables 3**-**S5**.

### Multicollinearity assessment

Multicollinearity analysis revealed severe collinearity between LDL-C and total cholesterol, as well as between AST, ALT, and the AST/ALT ratio (VIF > 5). To avoid model bias, LDL-C, AST, and ALT were excluded from subsequent multivariate analyses.

### Multivariate moderation analysis and independent predictors

**Figure 1:**
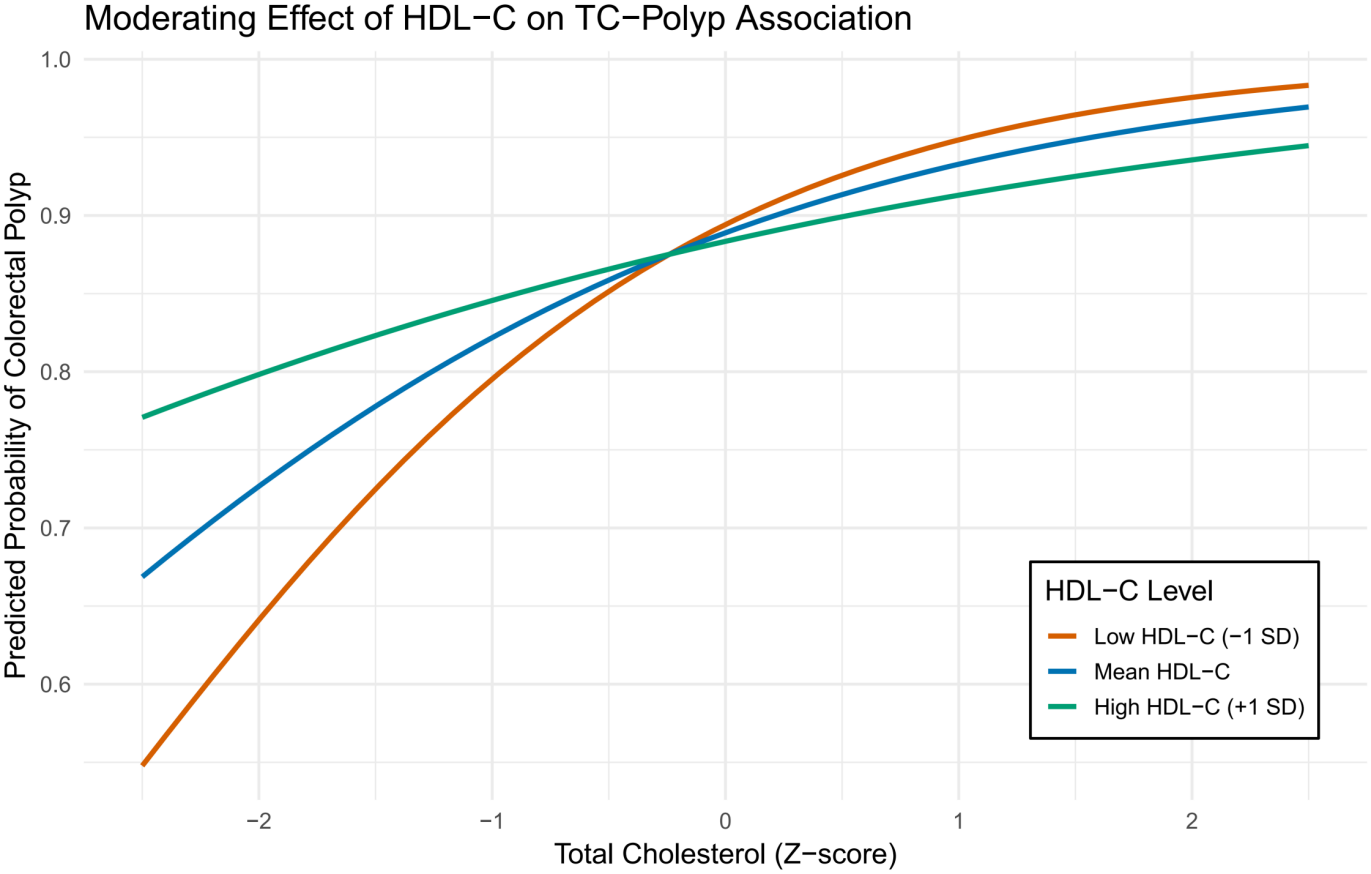
Suggestive negative moderating trend of HDL-C on the association between standardized TC and colorectal polyp risk. The risk gradient of TC was flattened at high HDL-C levels.

In our pre-specified multivariate logistic regression fully adjusted for age and all available baseline clinical covariates, a suggestive inverse moderating trend between standardized TC and HDL-C was observed (OR = 0.787, 95% CI: 0.626–0.994, P = 0.040, **Figure 1**). Elevated standardized TC independently increased the risk of colorectal polyps (OR = 1.570, 95% CI: 1.160–2.160, P = 0.004), whereas HDL-C exhibited no independent predictive effect after full covariate adjustment (OR = 1.150, 95% CI: 0.840–1.580, P = 0.394).

After full covariate adjustment, the main effect of HDL-C was statistically non-significant (OR = 1.150, P = 0.394), indicating isolated HDL-C levels could not independently predict polyp occurrence. However, the TC × HDL-C interaction term reached statistical significance (OR=0.787, P = 0.040), demonstrating HDL-C only functions by modifying the pro-polyp effect of total cholesterol. Specifically, elevated HDL-C buffered risk increments driven by high TC, while HDL-C concentrations made no difference in polyp risk among individuals with normal TC levels. The univariate comparison (**Supplementary Table 1**) further supported this pattern: baseline HDL-C levels were comparable between polyp and control groups (P = 0.495), so intergroup polyp disparities could not be attributed to raw HDL-C concentrations alone.

Age was mandatorily incorporated into the regression per our pre-planned analytical framework, despite lacking independent predictive power (OR = 0.989 per year of age, 95% CI: 0.966–1.010, P = 0.387). Despite the higher baseline age observed in controls, age showed no independent association with colorectal polyps after comprehensive covariate adjustment, suggesting the crude age gap between groups was driven by residual confounding rather than an inherent age-related polyp risk gradient.

**Figure 2:**
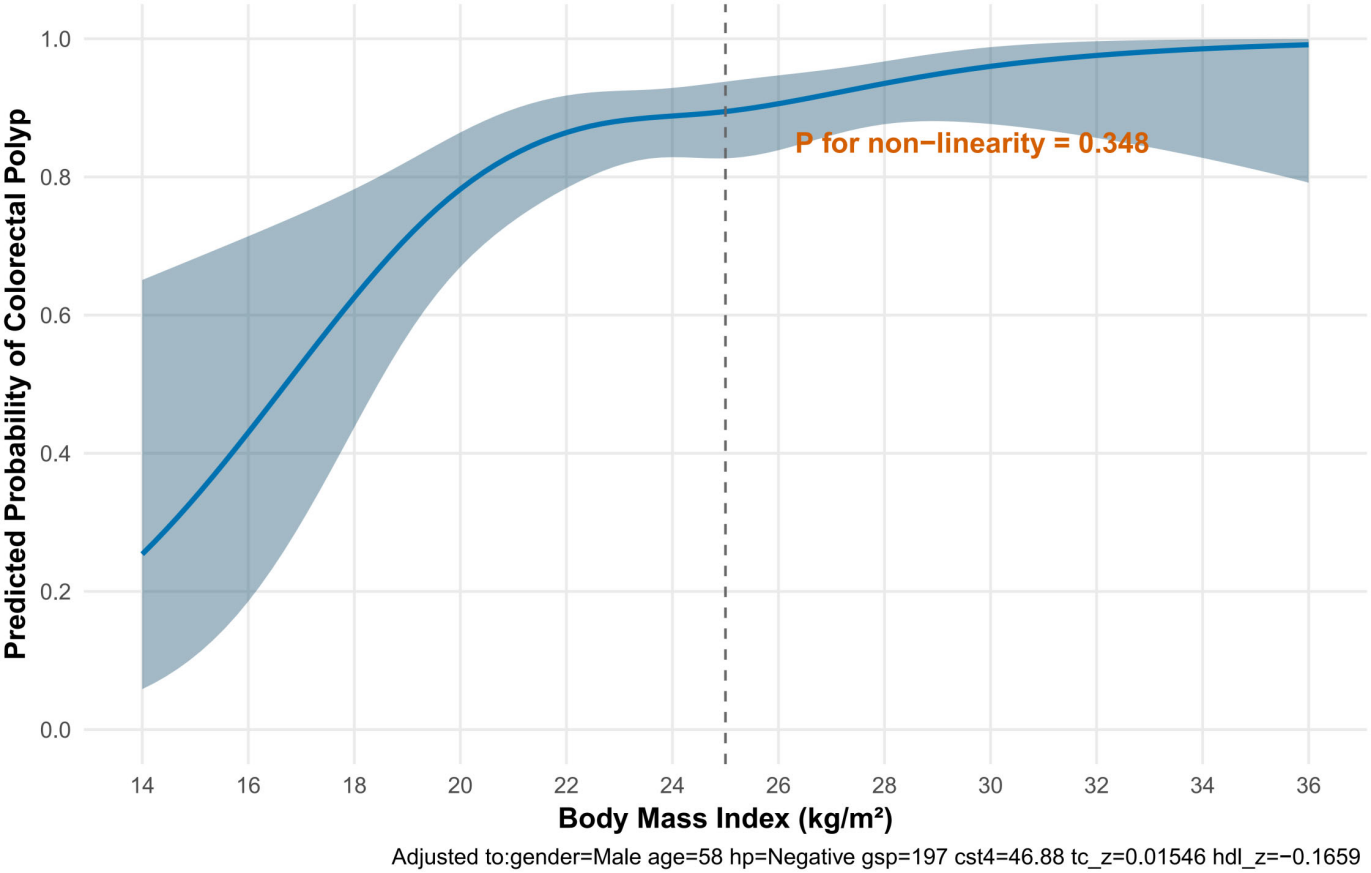
Restricted cubic spline curve for continuous BMI; P for non-linearity=0.348.

**Figure 3:**
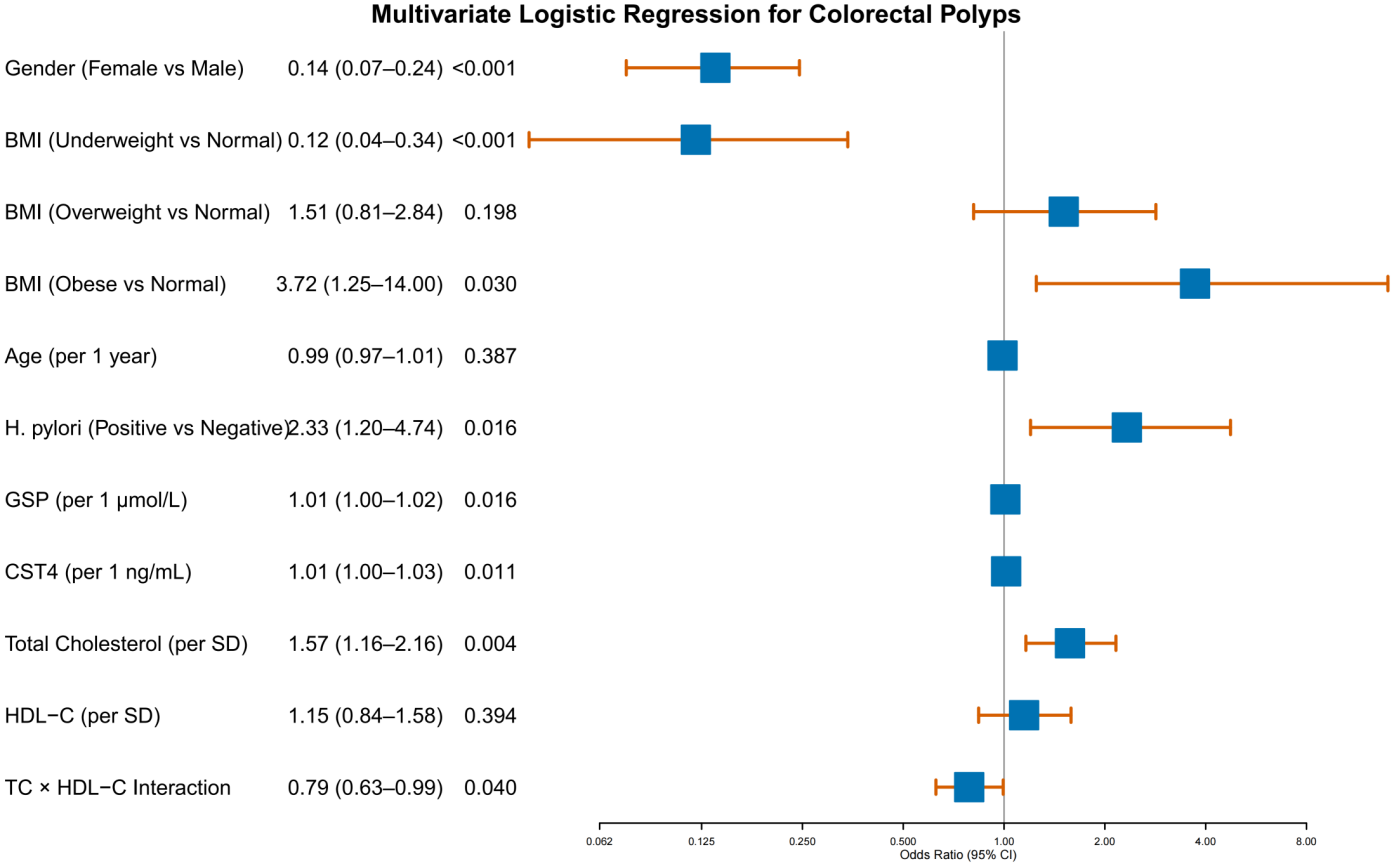
Multivariate logistic regression forest plot of fully adjusted primary model.

**Figure 4:**
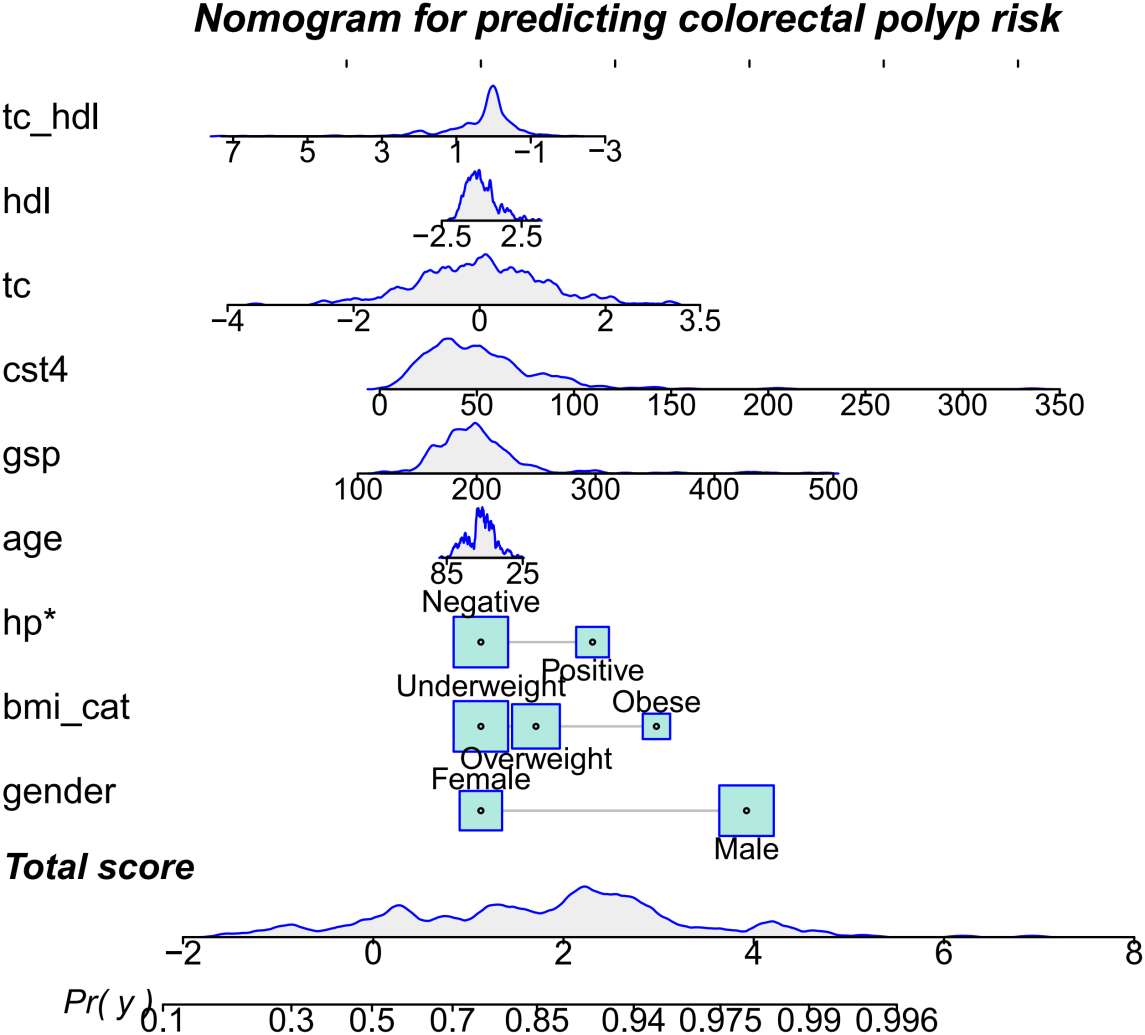
Nomogram for predicting colorectal polyp risk based on full logistic regression model including TC-HDL interaction term. The TC × HDL-C interaction term was reverse-coded for nomogram point assignment (i.e., higher scores assigned to lower TC × HDL-C product), consistent with the inverse interaction coefficient in **Table 2**. This recoding is for scoring purposes only and does not alter the regression model or its interpretation. Categorical axes are labeled with Female/Male, H. pylori Negative/Positive, and four BMI subgroups.

For categorical covariates, normal weight, male sex and Hp-negative patients served as unified reference groups. Compared with normal-weight individuals, obesity was associated with significantly higher polyp odds (OR = 3.720, 95% CI: 1.250–14.000, P = 0.030). Overweight showed no statistical correlation with polyp risk. Underweight was associated with lower polyp odds (OR = 0.120, 95% CI: 0.038–0.341, P<0.001), which further supports a positive correlation between adiposity and colorectal neoplasia. Restricted cubic spline analysis further verified the absence of a significant nonlinear association between continuous BMI and polyp incidence (P for non-linearity=0.348, **Figure 2**). Other independent risk predictors identified in this fully adjusted model included female sex (reference: male), which exerted a protective effect against polyps (OR=0.140, 95%CI: 0.070–0.240, P<0.01). Positive H. pylori status (reference: Hp-negative) was an independent risk factor for colorectal polyps (OR = 2.330, 95%CI: 1.200–4.740, P=0.016), elevated GSP (OR=1.010, 95%CI: 1.000–1.020, P=0.016) and elevated CST4 (OR=1.010, 95%CI: 1.001–1.030, P = 0.011). Full regression outputs are summarized in **Table 2** and visualized in **Figure 3**. The total points correspond to an estimated probability of colorectal polyp presence, ranging from <10% to >90%. (**Figure 4****).**

**Table 2:** Multivariate logistic regression analysis with moderation term for colorectal polyps.

Variable	β	SE	OR (95% CI)	P-value
Intercept	-1.85	1.21	0.157 (0.016–1.370)	0.101
Main effects				
TC (Z-score)	0.451	0.153	1.570 (1.160~2.160)	0.004
HDL-C (Z-score)	0.14	0.155	1.150 (0.840~1.580)	0.394
TC × HDL-C interaction term	-0.24	0.118	0.787 (0.626~0.994)	0.040
Other covariates (reference: normal weight/male/Hp negative)				
Age (per 1 year increase)	-0.011	0.012	0.989 (0.966~1.010)	0.387
BMI category: Underweight	-2.12	0.639	0.120 (0.038~0.341)	<0.001
BMI category: Overweight	0.412	0.626	1.510 (0.811~2.840)	0.198
BMI category: Obese	1.314	0.916	3.720 (1.250~14.000)	0.03
H. pylori infection (Positive)	0.846	0.342	2.330 (1.200~4.740)	0.016
GSP (per 1 µmol/L)	0.01	0.004	1.010 (1.000~1.020)	0.016
CST4 (per 1 ng/mL)	0.01	0.005	1.010 (1.000~1.030)	0.011
Gender: Female vs Male	-1.966	0.285	0.140 (0.070~0.240)	<0.001

**Figure 5:**
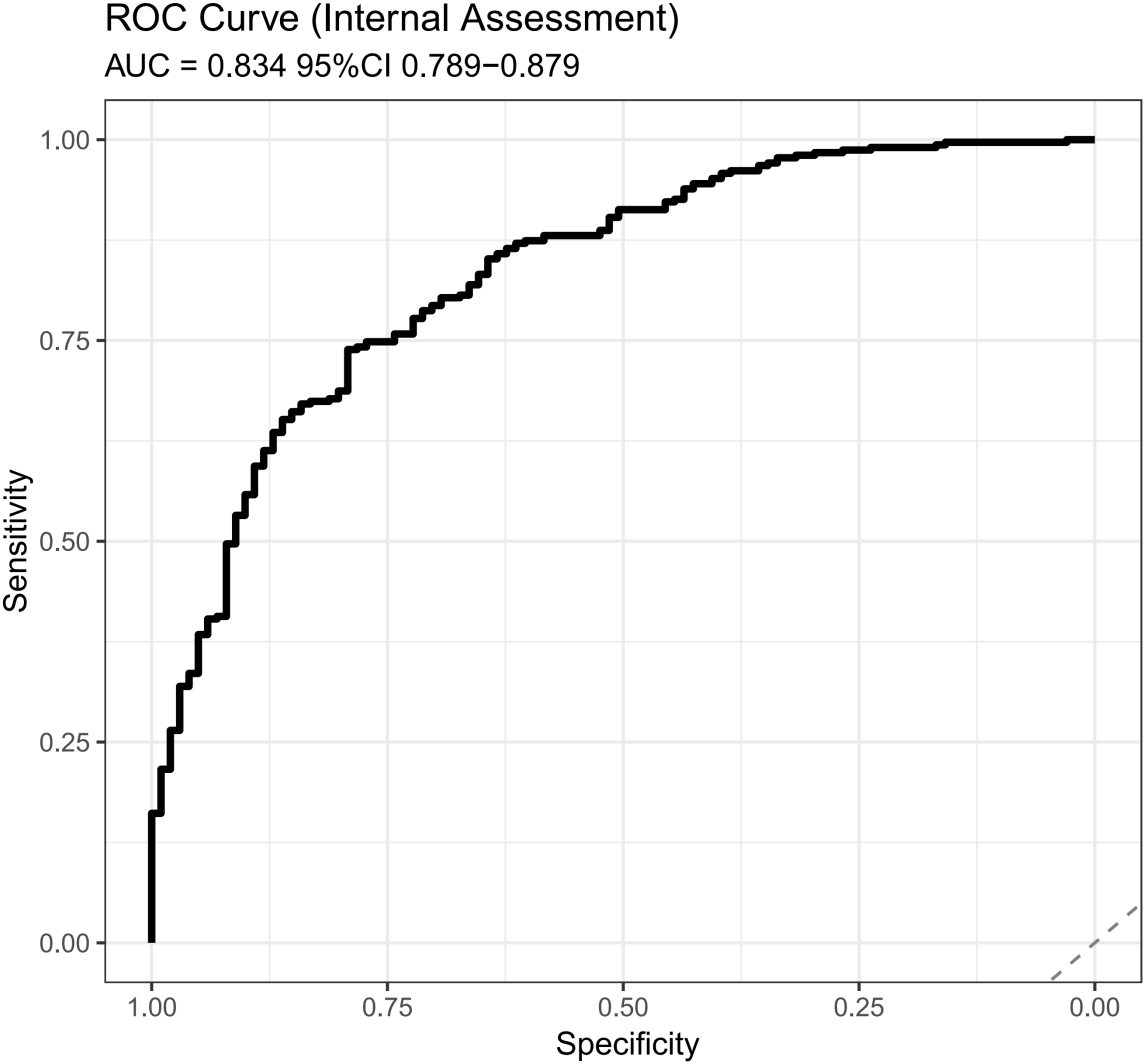
ROC curve of the predictive nomogram. AUC = 0.834 (95% CI: 0.789–0.879), n = 411.

**Figure 6:**
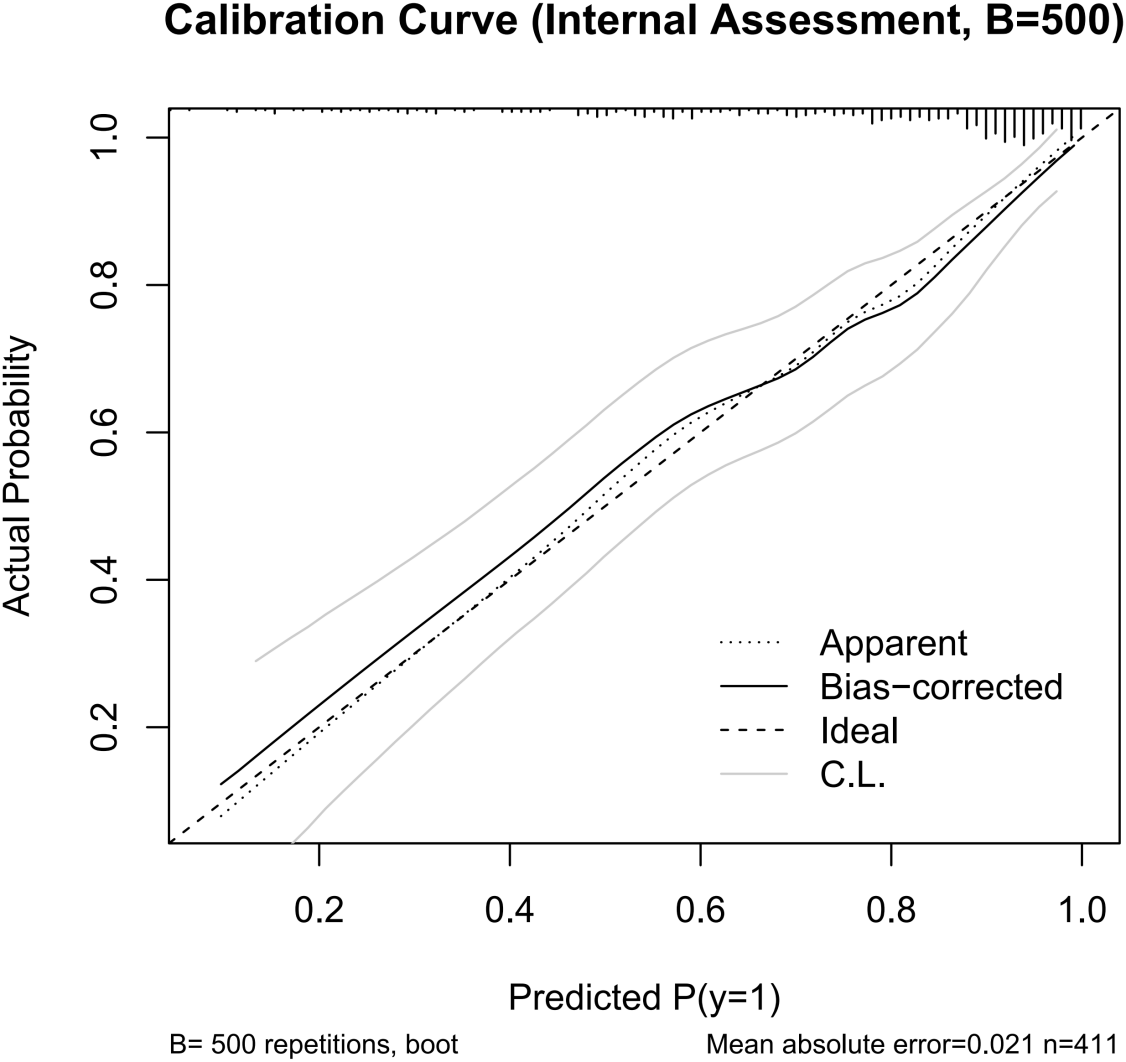
Bootstrap-calibrated calibration curve (B = 500). Mean absolute error = 0.021.

**Figure 7:**
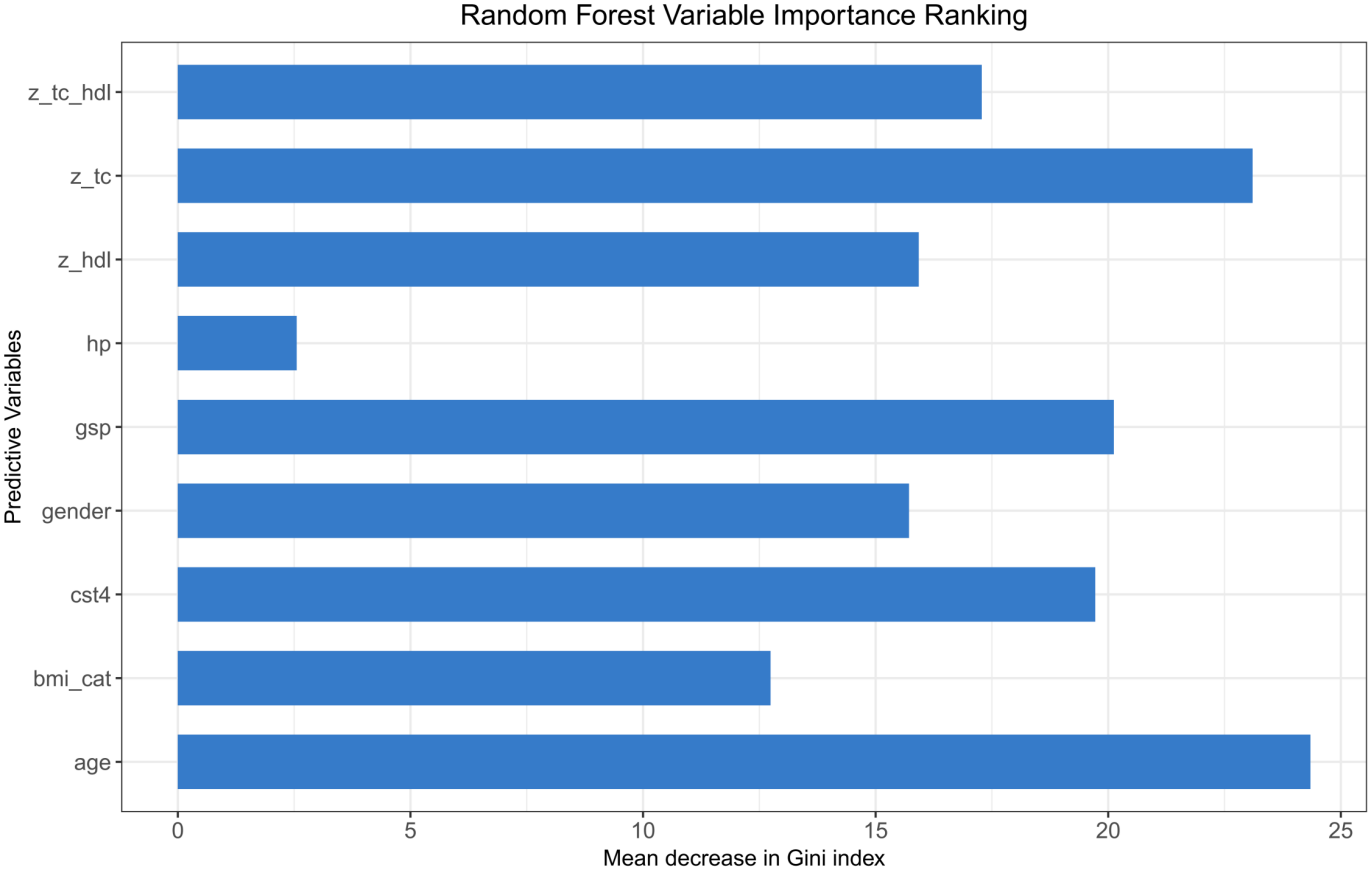
Random forest variable importance ranking; this ranking cannot independently verify the TC × HDL-C interaction effect.

The nomogram was constructed using the entire study population (n = 411). The model showed good discrimination with an AUC of 0.834 (95% CI: 0.789–0.879, **Figure 5**). Calibration curve with 500 bootstrap repetitions showed excellent agreement between predicted and actual probabilities, with a mean absolute error of 0.021 (**Figure 6**). Random forest analysis ranked age, standardized TC, GSP, CST4, and the TC × HDL-C interaction among variables with relatively high importance. Variable importance rankings reflect predictive weight within this cohort and should not be interpreted as independent validation of the interaction effect (**Figure 7**).

### Sensitivity analyses for lipid interaction

To exclude compositional coupling bias between TC and HDL-C, we performed a series of sensitivity analyses using alternative lipid measures (**Supplementary Table 6**). When non-HDL-C replaced TC, the non-HDL-C × HDL-C interaction maintained a consistent suggestive inverse trend (OR = 0.743, 95% CI:0.574–0.965, P = 0.023), suggesting that the observed moderating effect was not merely a mathematical artifact. In contrast, neither LDL-C (OR = 1.200, 95% CI:0.922–1.580, P = 0.178) nor the TC/HDL-C ratio (OR = 1.220, 95% CI:0.918–1.640, P = 0.172) showed significant associations with polyp risk.

### Subgroup analyses by polyp histology

Stratified analyses by polyp pathological subtype showed a consistent negative HDL-C moderating trend in both adenomatous and hyperplastic polyps at the descriptive level, though formal subgroup interaction testing was not performed due to limited subgroup sample sizes.

## Discussion

Our fully adjusted regression detected a suggestive inverse HDL-C moderating trend on the TC-polyp association after adjustment for age and clinical covariates, with consistent results observed in non-HDL-C sensitivity analyses. This provides a reasonable explanation for the clinical phenomenon that hypercholesterolemia does not uniformly increase polyp risk: higher HDL-C levels may attenuate the positive TC–polyp association at higher TC levels, potentially via anti-inflammatory and reverse cholesterol transport pathways.

In the multivariate model, total cholesterol was a significant risk factor (OR = 1.570, 95% CI: 1.160–2.160, P = 0.004), underscoring the inadequacy of evaluating TC alone. The protective effect of HDL-C in this context likely derives from its well-established physiological roles. First, HDL-C mediates reverse cholesterol transport, actively removing excess cholesterol from peripheral tissues, including the intestinal mucosa, thereby mitigating local lipid deposition and subsequent oxidative stress (22). Second, HDL particles possess potent anti-inflammatory properties capable of suppressing chronic, low-grade inflammation of the colonic epithelium—a recognized driver of the adenoma-carcinoma sequence (23, 24). Third, emerging evidence indicates that HDL enhances intestinal barrier function via upregulation of tight junction proteins, thereby reducing mucosal permeability and bacterial translocation, which are critical intermediate steps in cholesterol-induced epithelial proliferation (25). Our interaction plot visually suggests that at elevated HDL-C levels, the slope of the TC–polyp risk curve tends to flatten, though HDL-C did not exhibit a significant independent protective effect in this cohort. All participants were hospitalized patients with gastrointestinal discomfort requiring colonoscopy, rather than community screening crowds, which brings inherent selection bias and may interfere with serum lipid levels. Stratified analysis showed a consistent HDL-C protective tendency in both adenomatous and hyperplastic polyps. Given their distinct proliferative pathways, larger cohorts are needed to explore subtype-specific lipid effects in future research.

We constructed a predictive nomogram, whose discriminative performance was assessed by the ROC curve (**Figure 5**). This model integrates the TC × HDL-C interaction term alongside other independent predictors identified in multivariate analysis: sex, BMI category, H. pylori infection, glycated serum protein (GSP) and CST4. The nomogram enables clinicians to estimate a model-based probability within this inpatient cohort of harboring a colorectal polyp by summing weighted points without recourse to manual calculations. The model showed good discrimination with an AUC of 0.834 (95% CI: 0.789–0.879). Calibration was excellent using 500 bootstrap resampling, with a mean absolute error of 0.021, indicating close agreement between predicted probabilities and observed frequencies. The high variable importance of TC × HDL-C in random forest only indicates this interaction improves model discrimination among hospitalized patients. It cannot serve as independent evidence of HDL’s moderating effect, which was validated only by logistic interaction models and sensitivity tests.

The nomogram developed herein offers a pragmatic approach to risk stratification in colorectal polyp screening. Consider the following clinical scenario: a 55-year-old male patient presents for routine health assessment. He is overweight (BMI 26 kg/m²), has a positive *H. pylori* stool antigen test, a GSP of 210 µmol/L, a total cholesterol of 6.2 mmol/L, and an HDL-C of 0.9 mmol/L. Based on the nomogram, the cumulative point total would place him in a high-risk category, indicating a high predicted probability of harboring a colorectal polyp. This hypothetical scenario is illustrative only and is intended to demonstrate the potential clinical utility of the nomogram for risk estimation. However, because the nomogram was derived from a hospitalized inpatient cohort and has not been externally validated in independent or community-based populations, it should not be used as the sole determinant for endoscopic referral, nor should it be employed to defer indicated colonoscopy. Its potential role in optimizing endoscopic resource allocation remains hypothetical and requires prospective evaluation before any operational use can be recommended.

Several additional predictors warrant discussion. Female gender remained strongly protective (OR = 0.12, 95% CI: 0.06–0.24, P < 0.001), consistent with established epidemiology demonstrating higher polyp prevalence in men (26). Relative to the reference group of normal-weight participants, obesity was linked to higher polyp odds (OR = 3.720, 95% CI:1.250–14.000, P = 0.030). Underweight was associated with reduced polyp risk (P<0.001), whereas overweight showed no significant association, reinforcing the positive correlation between adiposity and colorectal neoplasia. H. pylori infection emerged as an independent risk factor (OR = 2.30, 95% CI: 1.18–4.50, P = 0.015), lending support to the hypothesis that chronic gastric infection may alter the gut microbiome and promote colonic inflammation (27). Glycated serum protein, a marker of short-term glycemic variability, exhibited a small but significant association (OR = 1.010 per µmol/L, 95% CI: 1.000–1.020, P = 0.016), underscoring the role of glucose dysmetabolism even in the absence of overt diabetes (28). CST4, a novel tumor marker, also demonstrated independent predictive value (OR = 1.010 per ng/mL, 95% CI: 1.000-1.030, P = 0.011), suggesting its potential utility in the early detection of colorectal lesions (29). By contrast, other tumor markers such as CA153, CA125, and CA724 did not retain independent predictive value after adjustment for metabolic variables, suggesting that their initial univariate associations were likely confounded by metabolic dysregulation.

Univariate analysis revealed differences in CA125 and CA724 between the polyp and control groups; however, these associations did not persist after multivariate adjustment (30). This observation is unsurprising. Both markers are glycoproteins that may be elevated in benign inflammatory conditions, including those associated with metabolic syndrome. Their initial significance was likely confounded by the metabolic variables incorporated into the final model. This finding underscores the importance of multivariate modeling: standalone tumor markers are insufficient for polyp screening and should be interpreted within the broader context of metabolic health. Furthermore, our stratified analyses (**Supplementary Tables 3**-**S5**) revealed that some metabolic indicators, particularly UA and AST/ALT, varied by polyp location, while most markers did not differ significantly by pathological subtype. These stratified findings may be consistent with a broad metabolic influence on the colonic mucosa rather than strict subtype-specific progression (31), but this interpretation remains speculative and requires confirmation in larger, well-powered cohorts. Exploratory stratified analyses showed descriptively similar moderating trends across hyperplastic and adenomatous subgroups; however, formal statistical testing for subgroup heterogeneity was not performed owing to limited sample sizes. These subgroup findings are preliminary and should not be interpreted as evidence of equivalent modifying effects across distinct histological pathways or as an indication of stage-specific involvement in the adenoma–carcinoma sequence (32). Confirmatory studies with larger, well-powered cohorts are needed before any subtype-related conclusions can be drawn.

### Limitations

This study has several notable limitations. First, all participants were hospitalized patients receiving colonoscopy for gastrointestinal complaints rather than community screening individuals, which may introduce selection bias and alter circulating serum lipid concentrations. Second, standardized records of key lifestyle confounders (smoking, alcohol intake, dietary habits, physical activity, family history of colorectal polyps and colonoscopy indications) were incomplete and inconsistent across inpatient charts, and these variables could not be incorporated into multivariate adjustment, potentially leading to residual confounding. Third, the predictive nomogram was built using the full study cohort with only bootstrap internal assessment; no independent external cohort was available to test its generalizability. Fourth, only total serum HDL-C concentrations were measured, without evaluation of HDL subfractions or functional HDL activity, which cannot fully capture HDL-mediated intestinal mucosal protective effects. Fifth, subgroup analyses stratified by polyp histology suffered from limited sample sizes, and we could not distinguish subtype-specific lipid moderating trends.

## Conclusion

After adjustment for age and baseline clinical covariates, we identified a suggestive inverse TC × HDL-C moderating trend, which remained robust in non-HDL-C sensitivity analysis. The internally assessed nomogram provides preliminary support for individualized colorectal polyp risk stratification. Study observations are limited by inpatient selection bias and incomplete adjustment for lifestyle risk factors. Large prospective community cohorts are needed to replicate these results and further investigate lipid moderating patterns across distinct polyp subtypes.

## Data Availability

The datasets analyzed in this study are not publicly available, nor included in the article or **Supplementary Materials**, due to patient privacy and ethical restrictions. De-identified data may be available from the corresponding author upon reasonable request, subject to approval by the Institutional Review Board of the Fourth Affiliated Hospital of Anhui Medical University. Requests to access these datasets should be directed to Zhengli Dou ch2004640011@fy.ahmu.edu.cn.

## Supplementary material

Supplementary Table 1 Univariate analysis of metabolic indicators.

Supplementary Table 2 Univariate analysis of tumor markers.

Supplementary Table 3 Metabolic indicators by polyp location.

Supplementary Table 4 Metabolic markers by polyp number (single vs. multiple).

Supplementary Table 5 compares metabolic indicators between hyperplastic and adenomatous polyp subgroups.

Supplementary Table 6 Sensitivity analysis of lipid interaction models.
