# Supplementary material for: HDL-C presents a suggestive negative moderating trend for the association between total cholesterol and colorectal polyps: model construction and bootstrap internal assessment of a predictive nomogram
Source: Front Endocrinol (Lausanne). 2026 Jul 22;17:1864105. doi: 10.3389/fendo.2026.1864105 (PMC13437356; doi:10.3389/fendo.2026.1864105)
Supplement: Supplementary file 1 [file Table1.docx]

| Metabolic indicators | Study group | Control group | t/Z | P |
| --- | --- | --- | --- | --- |
| TC | 4.82±0.91 | 4.43±1.12 | 3.47 | 0.001 |
| TG | 1.36(0.94,2.00) | 1.04(0.78,1.39) | -4.378 | ＜0.001 |
| LDL-C | 2.66±0.79 | 2.47±0.85 | 2.086 | 0.038 |
| HDL-C | 1.09(0.93,1.30) | 1.12(0.90,1.38) | -0.682 | 0.495 |
| FBG | 5.40(5.00,5.90) | 5.20(4.80,5.71) | -2.224 | 0.026 |
| GSP | 198(181,217) | 185(169,214) | -1.418 | 0.156 |
| UREA | 5.80(4.80,6.70) | 5.40(4.30,6.60) | -1.582 | 0.114 |
| UA | 340.00(279.00,393.00) | 291.00(248.00,338.00) | -5.112 | ＜0.001 |
| Cr | 62.10(50.80,70.10) | 57.00(47.00,67.00) | -2.449 | 0.014 |
| TBIL | 15.00(12.00,20.00) | 15.00(12.00,18.00) | -0.796 | 0.426 |
| TBA | 2.30(1.30,3.90) | 2.00(1.40,3.30) | -1.222 | 0.222 |
| AST/ALT | 1.05(0.81,1.31) | 1.20(1.00,1.52) | -4.326 | ＜0.001 |
