# Supplementary material for: HDL-C presents a suggestive negative moderating trend for the association between total cholesterol and colorectal polyps: model construction and bootstrap internal assessment of a predictive nomogram
Source: Front Endocrinol (Lausanne). 2026 Jul 22;17:1864105. doi: 10.3389/fendo.2026.1864105 (PMC13437356; doi:10.3389/fendo.2026.1864105)
Supplement: Supplementary file 2 [file Table2.docx]

| **Tumor markers** | **Study group** | **Control group** | **Z** | **P** |
| --- | --- | --- | --- | --- |
| CEA | 2.29 (1.64, 3.20) | 2.33 (1.50, 3.26) | -0.192 | 0.848 |
| CA125 | 10.80 (8.40, 15.10) | 9.50 (6.70, 12.30) | -3.341 | 0.001 |
| CA153 | 7.60 (5.80, 10.50) | 7.20 (5.40, 10.10) | -0.695 | 0.487 |
| CA199 | 5.80 (3.35, 10.74) | 5.63 (3.15, 10.27) | -0.533 | 0.594 |
| CA724 | 2.10 (1.44, 3.36) | 1.78 (1.12, 2.67) | -2.904 | 0.004 |
| CST4 | 49.05 (32.13, 68.00) | 42.90 (31.20, 60.30) | -1.853 | 0.064 |
