# Supplementary material for: HDL-C presents a suggestive negative moderating trend for the association between total cholesterol and colorectal polyps: model construction and bootstrap internal assessment of a predictive nomogram
Source: Front Endocrinol (Lausanne). 2026 Jul 22;17:1864105. doi: 10.3389/fendo.2026.1864105 (PMC13437356; doi:10.3389/fendo.2026.1864105)
Supplement: Supplementary file 3 [file Table3.docx]

| **Indicator** | **Left colon**  **(n=169)** | **Right colon**  **(n=31)** | **Transverse colon**  **(n=36)** | **Multiple locations**  **(n=74)** | **H/F** | **P** |
| --- | --- | --- | --- | --- | --- | --- |
| FBG | 5.30(5.00,5.80) | 5.60(5.15,6.50) | 5.50(5.10,6.07) | 5.45(5.10,6.00) | 6.518 | 0.089 |
| Cr | 62.20(50.70,70.10) | 58.00(51.30,68.00) | 66.10(58.00,77.45) | 59.85(50.38,70.00) | 2.992 | 0.393 |
| TG | 1.30(0.96,1.89) | 1.24(0.84,1.78) | 1.54(0.92,2.48) | 1.47(1.00,2.61) | 5.132 | 0.162 |
| HDL-C | 1.09(0.93,1.29) | 1.09(0.92,1.32) | 1.03(0.94,1.25) | 1.13(0.95,1.33) | 1.719 | 0.633 |
| UREA | 6.00(4.85,6.80) | 5.88(5.00,7.00) | 5.10(4.49,6.35) | 5.50(4.80,6.73) | 4.634 | 0.201 |
| UA | 336.00(271.00,388.00) | 299.00(255.00,361.00) | 380.00(324.00,415.50) | 360.50(283.25,416.75) | 13.692 | 0.003 |
| AST/ALT | 1.06(0.87,1.35) | 1.09(0.90,1.31) | 1.03(0.94,1.25) | 1.13(0.95,1.33) | 10.345 | 0.016 |
| TBIL | 15.00(12.13,20.00) | 14.80(11.90,18.50) | 15.50(12.50,21.25) | 14.60(11.80,19.30) | 3.785 | 0.286 |
| TBA | 2.20(1.30,3.70) | 3.10(1.45,4.75) | 2.00(1.30,4.00) | 2.65(1.42,4.25) | 3.257 | 0.354 |
| GSP | 198.00(181.00,217.00) | 205.00(185.00,229.00) | 212.50(188.00,235.75) | 208.00(183.25,232.50) | 8.340 | 0.039 |
| TC | 4.78±0.86 | 4.86±0.89 | 4.84±1.07 | 4.89±0.97 | 0.267 | 0.849 |
| LDL-C | 2.64±0.75 | 2.77±0.83 | 2.70±0.87 | 2.63±0.82 | 0.313 | 0.816 |
