# Supplementary material for: HDL-C presents a suggestive negative moderating trend for the association between total cholesterol and colorectal polyps: model construction and bootstrap internal assessment of a predictive nomogram
Source: Front Endocrinol (Lausanne). 2026 Jul 22;17:1864105. doi: 10.3389/fendo.2026.1864105 (PMC13437356; doi:10.3389/fendo.2026.1864105)
Supplement: Supplementary file 4 [file Table4.docx]

| **Metabolic indicators** | **Multiple polyps** | **Single polyp** | **T/Z** | **P** |
| --- | --- | --- | --- | --- |
| TC | 4.83±0.93 | 4.80±0.89 | 0.288 | 0.773 |
| TG | 1.415 (1.03, 2.00) | 1.28 (0.885, 1.995) | -1.671 | 0.095 |
| LDL-C | 2.475 (2.10, 3.15) | 2.67 (2.205, 3.14) | -1.134 | 0.257 |
| HDL-C | 1.11 (0.95, 1.31) | 1.09 (0.925, 1.30) | -0.547 | 0.584 |
| FBG | 5.40 (5.10, 6.00) | 5.40 (5.00, 5.90) | -0.403 | 0.687 |
| GSP | 198.00 (181.00, 216.00) | 197.50 (179.50, 217.50) | -0.269 | 0.788 |
| UREA | 5.70 (4.80, 6.60) | 5.85 (4.76, 6.80) | -0.358 | 0.72 |
| UA | 355.56±94.28 | 332.53±82.49 | 2.279 | 0.023 |
| Cr | 63.95 (51.70, 75.00) | 59.50 (50.00, 68.00) | -1.764 | 0.078 |
| TBIL | 15.00 (11.20, 20.00) | 15.00 (12.00, 20.00) | -0.283 | 0.777 |
| TBA | 2.35 (1.30, 4.00) | 2.30 (1.30, 3.75) | -0.344 | 0.973 |
| AST/ALT | 1.00 (0.806, 1.286) | 1.049 (0.836, 1.313) | -0.424 | 0.672 |
