# Supplementary material for: HDL-C presents a suggestive negative moderating trend for the association between total cholesterol and colorectal polyps: model construction and bootstrap internal assessment of a predictive nomogram
Source: Front Endocrinol (Lausanne). 2026 Jul 22;17:1864105. doi: 10.3389/fendo.2026.1864105 (PMC13437356; doi:10.3389/fendo.2026.1864105)
Supplement: Supplementary file 5 [file Table5.docx]

| **Metabolic indicators** | **Hyperplastic polyp group** | **Adenomatous polyp group** | **T/Z** | **P** |
| --- | --- | --- | --- | --- |
| TC | 4.92±0.97 | 4.81±0.91 | 0.695 | 0.488 |
| FBG | 5.60 (5.10, 6.26) | 5.35 (5.00, 5.90) | -1.351 | 0.177 |
| GSP | 197.00 (174.00, 219.00) | 198.00 (181.00, 216.00) | -0.389 | 0.697 |
| TG | 1.30 (1.04, 2.08) | 1.365 (0.94, 1.995) | -0.16 | 0.873 |
| HDL | 1.185 (0.96, 1.31) | 1.09 (0.93, 1.30) | -1.18 | 0.238 |
| LDL | 2.62 (2.20, 3.16) | 2.60 (2.125, 3.135) | -0.073 | 0.942 |
| Urea | 6.00 (4.50, 6.80) | 5.75 (4.80, 6.70) | -0.277 | 0.782 |
| UA | 358.50 (271.00, 416.00) | 339.50 (281.50, 392.00) | -0.416 | 0.678 |
| Cr | 59.50 (50.00, 65.50) | 62.70 (50.90, 72.80) | -1.804 | 0.071 |
| AST/ALT | 1.00 (0.807, 1.250) | 1.048 (0.816, 1.313) | -0.329 | 0.743 |
| TBIL | 14.50 (11.00, 16.00) | 15.00 (12.00, 20.00) | -1.587 | 0.113 |
| TBA | 2.25 (1.30, 3.60) | 2.30 (1.30, 4.00) | -0.616 | 0.538 |
