# Supplementary material for: HDL-C presents a suggestive negative moderating trend for the association between total cholesterol and colorectal polyps: model construction and bootstrap internal assessment of a predictive nomogram
Source: Front Endocrinol (Lausanne). 2026 Jul 22;17:1864105. doi: 10.3389/fendo.2026.1864105 (PMC13437356; doi:10.3389/fendo.2026.1864105)
Supplement: Supplementary file 6 [file Table6.docx]

| Model | Variable | OR (95% CI) | P |  |
| --- | --- | --- | --- | --- |
| Primary model | Standardized TC | 1.570 (1.160–2.160) | 0.004 |  |
|  | Standardized HDL-C | 1.150 (0.840–1.580) | 0.394 |  |
|  | TC × HDL-C interaction | 0.787 (0.626–0.994) | 0.04 |  |
| Sensitivity 1 (non-HDL-C) | Standardized non-HDL-C | 1.570 (1.180–2.110) | 0.002 |  |
|  | Standardized HDL-C | 1.200 (0.891–1.620) | 0.236 |  |
|  | non-HDL-C × HDL-C interaction | 0.743 (0.574–0.965) | 0.023 |  |
| Sensitivity 2 (LDL-C) | Standardized LDL-C | 1.200 (0.922–1.580) | 0.178 |  |
| Sensitivity 3 (TC/HDL ratio) | Standardized TC/HDL-C ratio | 1.220 (0.918–1.640) | 0.172 |  |
|  |  |  |  |  |
